# High Hepatitis E Seroprevalence Among Displaced Persons in South Sudan

**DOI:** 10.4269/ajtmh.16-0620

**Published:** 2017-06-07

**Authors:** Andrew S. Azman, Malika Bouhenia, Anita S. Iyer, John Rumunu, Richard Lino Laku, Joseph F. Wamala, Isabel Rodriguez-Barraquer, Justin Lessler, Etienne Gignoux, Francisco J. Luquero, Daniel T. Leung, Emily S. Gurley, Iza Ciglenecki

**Affiliations:** 1 Department of Epidemiology, Johns Hopkins Bloomberg School of Public Health, Baltimore, Maryland; 2 Médecins Sans Frontières, Geneva, Switzerland; 3 World Health Organization, Geneva, Switzerland; 4 Division of Infectious Diseases, Department of Internal Medicine, University of Utah School of Medicine, Salt Lake City, Utah; 5 South Sudan Ministry of Health, Juba, South Sudan; 6 World Health Organization, Juba, South Sudan; 7 Epicentre, Paris, France; 8 Department of International Health, Johns Hopkins Bloomberg School of Public Health, Baltimore, Maryland; 9 International Centre for Diarrhoeal Disease Research, Bangladesh (icddr,b), Dhaka, Bangladesh

## Abstract

Large protracted outbreaks of hepatitis E virus (HEV) have been documented in displaced populations in Africa over the past decade though data are limited outside these exceptional settings. Serological studies can provide insights useful for improving surveillance and disease control. We conducted an age-stratified serological survey using samples previously collected for another research study from 206 residents of an internally displaced person camp in Juba, South Sudan. We tested serum for anti-HEV antibodies (IgM and IgG) and estimated the prevalence of recent and historical exposure to the virus. Using data on individuals' serostatus, camp arrival date, and state of origin, we used catalytic transmission models to estimate the relative risk of HEV infection in the camp compared with that in the participants' home states. The age-adjusted seroprevalence of anti-HEV IgG was 71% (95% confidence interval = 63–78), and 4% had evidence of recent exposure (IgM). We estimated HEV exposure rates to be more than 2-fold (hazard ratio = 2.3, 95% credible interval = 0.3–5.8) higher in the camp than in the participants' home states, although this difference was not statistically significant. HEV transmission may be higher than previously appreciated, even in the absence of reported cases. Improved surveillance in similar settings is needed to understand the burden of disease and minimize epidemic impact through early detection and response.

## Introduction

Hepatitis E virus (HEV) is thought to be responsible for over 3 million symptomatic cases of acute hepatitis and more than 50,000 deaths each year worldwide.^1^,^2^ Though data from most of the world is scarce, a growing body of evidence suggests that this acute viral infection may be responsible for up to 10% of maternal deaths in some areas like Bangladesh.^3^,^4^ Over the past decade, large outbreaks of HEV have been reported in displaced populations in east Africa, including a large outbreak in northern Uganda responsible for more than 10,000 cases of acute jaundice and a 2% case fatality ratio (CFR), and one in South Sudan with more than 5,000 cases and a CFR in excess of 10% among pregnant women.^5^,^6^

While access to safe water and improved sanitation will halt most, if not all, HEV (genotypes 1 and 2) transmission, water and sanitation interventions, including chlorine disinfection, in low-resource settings have proven less effective than anticipated in reducing the risk of HEV infection.^7^ It is possible that poor/inappropriate compliance with these interventions is the reason they have not been effective, but this disease is difficult to combat with conventional approaches. Fortunately, a recombinant vaccine, HEV239 (Hecolin^®^; Innovax, Xiamen, China), has been shown to be highly efficacious in reducing the incidence of clinical HEV in a large randomized clinical trial in China.^8^ This vaccine is not currently World Health Organization (WHO) prequalified; however, the WHO recommends that it be considered for use in outbreak settings.^9^ The vaccine has a three-dose schedule, with the third dose given 6 months after the first, making it less than ideal for outbreak response, although data from the clinical trial suggest that reduced (one or two) dose schedules may be highly protective.^8^

Insensitive surveillance systems, combined with a high proportion of infected persons being asymptomatic and mildly symptomatic, contribute to our poor understanding of HEV epidemiology in resource-poor settings. This is especially true in Africa, where the bulk of evidence comes from a handful of large outbreaks over the past 10 years in Uganda, Sudan, South Sudan, and surrounding countries.^5^–^7^,^10^,^11^ New insights into the epidemiology of this disease, especially in settings where the vaccine may be used in an outbreak, may help provide important clues regarding how and where the vaccine may have the greatest impact.

In South Sudan, confirmed HEV has been reported from throughout the country over the past 10 years with most cases reported from camps of displaced people, including an epidemic that started in 2015.^6^,^12^,^13^ Outbreaks may be more frequently reported from camps because HEV transmission is higher in camps than in communities due to overcrowding and often poor water and sanitation conditions. Alternatively, the increased reporting from camps may simply reflect the strength of surveillance systems in camps compared with communities in the country and differential awareness of this underappreciated disease.

Herein, we report results from a serological survey, using samples originally collected for a cholera immunogenicity study,^14^ among internally displaced persons (IDPs) in Juba, South Sudan, to characterize patterns of historical exposure to HEV and to better understand the relative risk of exposure within the camp compared with the communities from where participants were displaced.

## Methods

### Study setting.

This study took place in the United Nations House protection of civilians camp in Juba, South Sudan, which at the time housed more than 30,000 IDPs. When ethnically motivated violence erupted in December 2013, protection of civilian camps and other informal IDP camps emerged and hosted people fleeing the threat of violence from both near and far. Though the conditions within the camp had improved since the start of the conflict, they remained only at the threshold of acceptability in humanitarian emergencies nearly 2 years later, with only approximately 15 L of safe drinking water per person per day and over 20 people sharing each latrine.^15^

### Selection and enrollment of participants.

This study used samples collected from an oral cholera vaccine immunogenicity study,^14^ where individuals older than 1 year were recruited from vaccination posts (and in some instances households) during a mass oral cholera vaccination campaign in June–July 2015. We enrolled an age-stratified convenience sample of participants who were willing to provide blood on that day and expressed willingness to return for additional follow-up visits (though only the first visit samples are used here). All participants (and/or their guardians) provided written informed consent to participate in the study including consent to use remaining samples for research on other enteric pathogens like HEV.

### Data collection.

After providing informed consent, trained study staff administered an electronic questionnaire to participants collecting basic demographic data in addition to details on where each person had resided before coming to the camp and when he/she had arrived. After completion of the questionnaire, a trained study nurse or phlebotomist drew 3–5 mL of venous blood into serum-separating vacutainers. Within 12 hours, study laboratory technicians centrifuged the blood and extracted serum. Serum was then stored at −20°C or below until it was shipped on dry ice to the reference laboratory at the University of Utah.

Testing of these specimens for HEV was within the original scope of consent and was approved by the Johns Hopkins Bloomberg School of Public Health and The Republic of South Sudan ethical review boards.

### Laboratory methods.

Serum samples were tested for the presence of anti-HEV IgG and IgM using an immunoassay kit (Wantai HEV IgG [WE-7296], IgM [WE-7196] ELISA kits; Beijing Wantai Biological Pharmacy Enterprise Co. Ltd., Beijing, China). Samples with a standardized optical density > 1.1 were considered positive, those < 0.9 were considered negative, and those in the range 0.9–1.1 were considered indeterminate according to the manufacturer's instructions.

### Statistical analyses.

We estimated the age-adjusted seroprevalence using poststratification weights based on the population age distribution from camp registration data collected the same month as the data collection (International Office of Migration, internal report). Wald-type 95% confidence intervals (CIs) were estimated taking into account this adjustment using the “confint” command in R's survey package.^16^,^17^ We compared the relative risk of IgG positivity between different groups and estimated CIs as the 2.5th and 97.5th quantiles of 1,000 (age-adjusted) bootstrap estimates.^17^

To determine whether being in the camp conferred any increased HEV infection risk compared with that in individuals' state of origin, we took two approaches ranging in complexity. First, we simply compared the age-adjusted seroprevalence of those who arrived in the camp in the first “wave” of displacements (on or before January 1, 2014, *N* = 98) to those who arrived within the 6 months before the survey (after January 1, 2015, *N* = 59), with the hypothesis being that those who have been in the camp the longest would have higher anti-HEV seroprevalence if there was indeed transmission in the camp.

### Catalytic transmission models.

Second, to compare the force of infection (i.e., the rate per capita at which susceptible individuals become infected, or hazard) in the camps to that in the community where the displaced individuals came from, a more direct measure of relative risk, we used the age-stratified and location of origin–stratified seroprevalence curves to fit catalytic transmission models.^18^ These models, which have been used to estimate the historical force of infection for other diseases,^18^–^20^ assume that seropositivity is a saturating state and no IgG-positive individual will ever again be at risk of infection.

The heterogeneity in arrival times of people of different ages from different locations provides the information needed to estimate the historical average forces of infection inside and outside the camp. The probability that an individual was IgG seropositive at the time of the study given the participants age (*a*), his/her home state (*s*), and the time he/she spent in the camp (*t*_c_, in 4-month intervals) was modeled as:

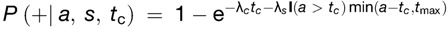
where, λ_s_ represents the 4-month cumulative hazard of infection in the state of origin, λ_c_ the 4-month cumulative hazard of infection in the camp, and *t*_max_ represents the maximum historical time of exposure in a participant's home state that can contribute to these estimates (assumed to be 25 years in the main analyses given the limited sample size and minimal evidence of transmission decades ago^21^ [see Supplemental Table 1 for alternative assumptions]).We used two different variants of this model, one where the average 4-month cumulative hazard of infection from each state of origin was assumed equal (i.e., all λ_s_'s were equal) and another where each state had its own independent value.

We used a Bayesian framework and the Stan programming language to estimate model parameters and assumed that each observation came from a Bernoulli distribution with the probability of positivity, as shown above. The hazards of infection were estimated on the logit scale with diffuse Gaussian priors (variance = 1,000) centered at a mean of zero. We drew samples from the joint posterior distribution using the standard built-in samplers in the RStan package in R.^22^ We ran four parallel chains, each with 7,000 iterations, and assessed convergence visually and through use of the Gelman-Rubin (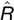) statistic.^22^ The estimates presented represent the posterior means, and the 95% credible intervals (CrIs) are the 2.5th and 97.5th quantiles from the posterior distributions after a burn-in period of 3,500 draws.

## Results

We enrolled 206 people between 1 and 59 years of age, though most participants were older children and adults and 131 (64%) were female. All participants were internally displaced people who had arrived in the camp between December 15, 2013 and May 7, 2015, fleeing from four different states: Central Equatoria, Jonglei, Upper Nile, and Unity States of South Sudan.

### Seroprevalence.

**Figure 1.**
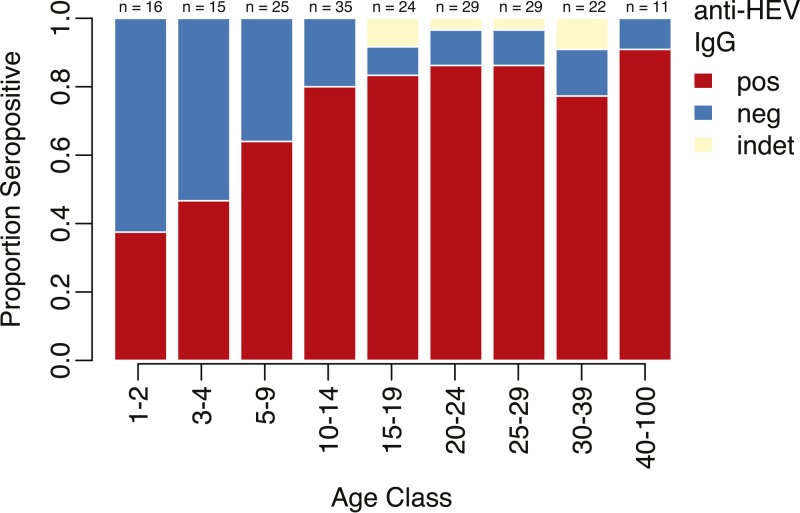
Seroprevalence of anti-hepatitis E virus (HEV) IgG by age group. Red represents the proportion positive, blue represents those negative, and yellow represents indeterminate readings.

Six samples had IgG levels that were considered indeterminate and were (conservatively) considered negative, and 75% (154/206) were positive for anti-HEV IgG. Since these samples did not come from a representative sample of the camp population, they do not reflect the true seroprevalence in the population. Using direct adjustment methods, we estimated the age-adjusted seroprevalence of HEV IgG to be 71% (95% CI = 63–78). In general, we saw increasing seroprevalence with age (*P* = 0.0002 for linear relationship, Figure 1

and Supplemental Figure 1 and Table 2), with consistently higher seroprevalence among females compared with males (Supplemental Figure 2), although these differences were not significant.

Although there had been no clinical cases of HEV detected within the camp, we found nine samples (4.4% adjusted and crude prevalence) positive for anti-HEV IgM, all of whom were also IgG positive. One additional sample was indeterminate for anti-HEV IgM. The IgM-positive individuals came from Unity State (*N* = 5), Central Equatoria State (*N* = 3), and Jonglei State (*N* = 1) and reported arriving to the camp 0.4–18 months before the start of the study (interquartile range = 4.6–18.7 months).

**Figure 2.**
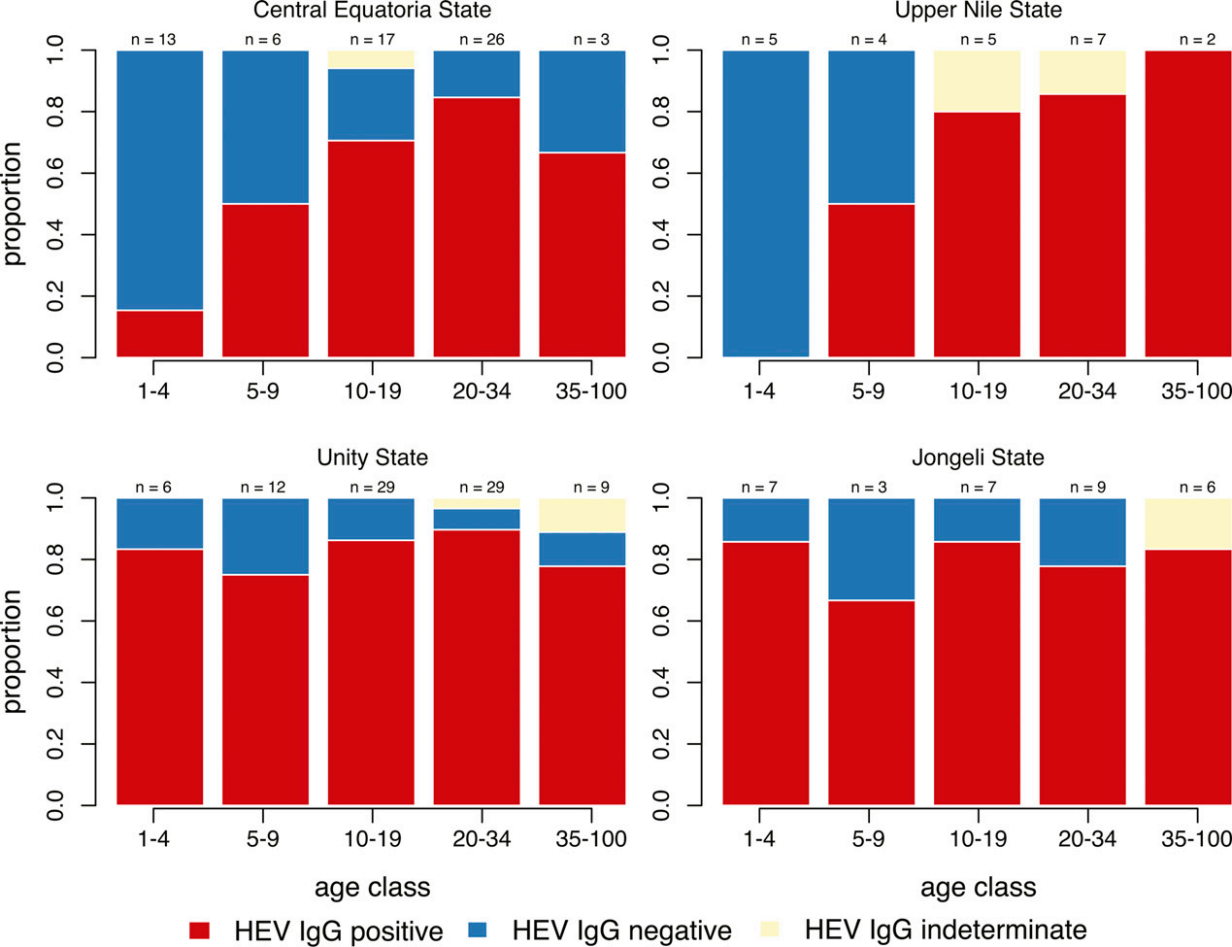
Seroprevalence of anti-hepatitis E virus (HEV) IgG by age group and state of origin. Red represents the proportion positive, blue represents those negative, and yellow represents indeterminate readings.

While the overall seroprevalence increased by age (Figure 1 and Supplemental Figure 1), the study population represents a mix of people with potentially different historical exposure to HEV. Stratifying by location (i.e., state) of origin, we found different seroprevalence patterns by age, depending on location of origin (Figure 2

and Supplemental Figure 3). In those coming from Central Equatoria and Upper Nile States, seroprevalence increased with age, whereas in those coming from Unity and Jonglei States, we found similar seroprevalence across most (or all) the age groups. Seroprevalence in those coming from Central Equatoria was 63%, 61% from Upper Nile, 85% from Unity, and 81% from Jonglei.

### Exposure risk in the camp.

Combining individuals from all home states, we found that those who had been in the camp for at least 1.5 years had a 3% increased risk of being IgG seropositive compared with those who arrived within the 6 months before the study, though this is likely confounded by age and location of origin. To account for potential differences in historical exposure, we stratified by participants' home state and found a higher risk of being seropositive (42% increase in Central Equatoria, 79% increase in Jonglei, 25% increase in Upper Nile) in those who initially arrived in the camp (on or before January 1, 2014) compared with those who had come in the previous 6 months (after January 1, 2015), except for those coming from Unity State (8% less).

To gain further insight into the age-adjusted risk of HEV exposure in the camp compared with what the risk would have been had they remained in their home state, we constructed a catalytic transmission model to estimate the force of infection in the camp and in each of the four home states. We estimated a 2.4- to 5.3-fold increase in the hazard of HEV in the camp compared with that estimated for Central Equatoria State (hazard ratio [HR] = 5.3, 95% CrI = 0.8–15.0), Jonglei State (HR = 3.6, 95% CrI = 0.5–10.1), Upper Nile State (HR = 4.4, 95%CrI = 0.6–13.4), and Unity State (HR = 2.4, 95% CrI = 0.6–13.4), although none were statistically significant. In an even simpler model, assuming all home states had the same average historical 4-month hazard of HEV exposure, we estimated a 2.3 (95% CrI = 0.3–5.8) times higher force of infection in the camp compared with the participants' previous residence. Within this simple model, we estimate that 6.5% (95% CrI = 1–14.3%) of susceptible individuals will have been exposed over each 4-month period in the camp. Analyses with alternative assumptions yielded similar qualitative results, although the HR decreased as we reduced the assumed number of years that HEV had circulated in participants' home states (*t*_max_, Supplemental Table 2).

## Discussion

In this study, we found a high seroprevalence of anti-HEV (IgG) antibodies and evidence of recent exposure (IgM) in a population of internally displaced people in South Sudan. Part of this seroprevalence could be driven by recent transmission in the states where the study participants originally came from, for example, from Unity State, where an epidemic had been ongoing.^13^ However, our results suggest that the risk of HEV infection in the displaced person camp may have been, on average, higher than in their home states, despite the absence of documented clinical cases of acute jaundice (nor confirmed HEV) within the camp.

HEV seroprevalence estimates from Africa vary widely though seroprevalence estimates of over 60% within the general population have not been found frequently, even during or after large outbreaks.^5^,^23^ Even in “hyperendemic” areas, including Nepal and Bangladesh, documented seroprevalence has typically been estimated at levels well below those in this study.^24^ Our results suggest that in South Sudan, there is more transmission than was previously thought with potentially many unrecognized deaths related to the disease.

In displaced person camps, people from places with different historical epidemiologic profiles come together and reside in close quarters where conditions for disease spread are high. In this study, subpopulations from each of the four home states had different seroprevalence, with two age-specific patterns emerging (Figure 2); first, a profile where seroprevalence rises with age, indicative of endemic circulation of the virus; second, constant seroprevalence by age, suggestive of a recent outbreak. This mix can result in a population-level seroprevalence much different from that of any one location. If seroprevalence is correlated with protection, this may leave populations who were once protected by herd immunity in their home state, residing in a camp with the potential for a large outbreak. When assessing epidemic threat in mixed populations, like in the camp in Juba, the different epidemiologic contexts where people came from should be considered.

This study comes with several limitations. First, our sample size was small, so that inference on subgroups, such as age and state of origin, can only be made with limited precision. Our sample was a convenience sample; therefore, participants may have differed in some ways from the general population in factors related to HEV risk. Our classification by previous state of residence is unlikely to be specific enough to categorize people's historical exposure to HEV, both because we do not know if they had spent their entire life in that state (e.g., they could have been in another camp setting before) and due to variation in HEV exposure risk within a state. Lastly, our models estimating the increased risk within the camp are simplificaions of a complex history of human–pathogen interactions. For example, we estimated the average 4-month hazard of HEV exposure over the preceding 25 years in each participant's home state, which is likely comprised with periods of both high and low exposure incidence. In sensitivity analyses, we found that shortening the time window to as low as 15 years attenuated estimates of the HR comparing risk in the camp to the home states, although the point estimates remained above one. Future studies with a larger sample size and more detailed data could extend and improve these models by accounting for known and unknown historical patterns in transmission intensity.

The individuals who were IgM positive may not have been indicative of transmission in the camp, as they may have been exposed before arriving at the camp, or may have been false positives. Some previous studies suggest that IgM wanes to undetectable levels within 3–4 months,^25^,^26^ whereas others have demonstrated that this may be as long as 1 year for some individuals.^25^,^27^,^28^ Most of the IgM-positive participants had been in the camp for longer than 6 months, with some up to 1.5 years, suggesting it is unlikely they had been exposed before coming. Given the high specificity of the IgM assay used (> 99%), even in settings with low HEV prevalence, it is highly unlikely to have nine false positives with the sample size of this study.^29^ Further laboratory testing for viremia, which has a shorter half-life than IgM,^30^ could shed more light on the timing and location of these exposures.

Large protracted outbreaks have occurred in South Sudan, particularly in camps, and our study provides new insights into the dynamics of HEV transmission both in the camps and across the country. When populations of susceptible people enter camps with others coming from areas with endemic transmission (or an ongoing outbreak), camp conditions provide ample opportunities for hepatitis E epidemics to explode. Although it has not been used to date outside of China as a public health tool, hepatitis E vaccine could be used to reduce the risk of outbreaks within this context. However, given the logistical constraints of the current three-dose regimen, vaccination campaigns must act quickly, if not preemptively, and not wait until large outbreaks are already underway. There is hope that among those with previous exposure to HEV, a shortened two-dose regimen may be efficacious,^31^ thus allowing more adaptive outbreak response with vaccine. However, more evidence from field studies or clinical trials is needed to understand the utility of reduced dose regimens.

Our study illustrates that HEV infections may be more common than previously appreciated, suggesting the possibility of a higher (yet undetected) burden even outside of reported outbreaks. Enhanced surveillance for HEV can help improve our understanding of this disease and, if more clinical cases are detected, it may strengthen the case to provide more attention and resources to this vaccine-preventable disease. In settings like South Sudan where large outbreaks have occurred and evidence of high historical exposure exists, more efforts should be put into the prevention and control of this neglected disease to reduce future morbidity and mortality.

## Supplementary Material

Supplemental Datas.

## References

[1] Lozano R, Naghavi M, Foreman K, Lim S, Shibuya K, Aboyans V, Abraham J, Adair T, Aggarwal R, Ahn SY, Alvarado M, Anderson HR, Anderson LM, Andrews KG, Atkinson C, Baddour LM, Barker-Collo S, Bartels DH, Bell ML, Benjamin EJ, Bennett D, Bhalla K, Bikbov B, Bin Abdulhak A, Birbeck G, Blyth F, Bolliger I, Boufous S, Bucello C, Burch M, Burney P, Carapetis J, Chen H, Chou D, Chugh SS, Coffeng LE, Colan SD, Colquhoun S, Colson KE, Condon J, Connor MD, Cooper LT, Corriere M, Cortinovis M, de Vaccaro KC, Couser W, Cowie BC, Criqui MH, Cross M, Dabhadkar KC, Dahodwala N, De Leo D, Degenhardt L, Delossantos A, Denenberg J, Des Jarlais DC, Dharmaratne SD, Dorsey ER, Driscoll T, Duber H, Ebel B, Erwin PJ, Espindola P, Ezzati M, Feigin V, Flaxman AD, Forouzanfar MH, Fowkes FG, Franklin R, Fransen M, Freeman MK, Gabriel SE, Gakidou E, Gaspari F, Gillum RF, Gonzalez-Medina D, Halasa YA, Haring D, Harrison JE, Havmoeller R, Hay RJ, Hoen B, Hotez PJ, Hoy D, Jacobsen KH, James SL, Jasrasaria R, Jayaraman S, Johns N, Karthikeyan G, Kassebaum N, Keren A, Khoo JP, Knowlton LM, Kobusingye O, Koranteng A, Krishnamurthi R, Lipnick M, Lipshultz SE, Ohno SL, Mabweijano J, MacIntyre MF, Mallinger L, March L, Marks GB, Marks R, Matsumori A, Matzopoulos R, Mayosi BM, McAnulty JH, McDermott MM, McGrath J, Mensah GA, Merriman TR, Michaud C, Miller M, Miller TR, Mock C, Mocumbi AO, Mokdad AA, Moran A, Mulholland K, Nair MN, Naldi L, Narayan KM, Nasseri K, Norman P, O'Donnell M, Omer SB, Ortblad K, Osborne R, Ozgediz D, Pahari B, Pandian JD, Rivero AP, Padilla RP, Perez-Ruiz F, Perico N, Phillips D, Pierce K, Pope CA, Porrini E, Pourmalek F, Raju M, Ranganathan D, Rehm JT, Rein DB, Remuzzi G, Rivara FP, Roberts T, De León FR, Rosenfeld LC, Rushton L, Sacco RL, Salomon JA, Sampson U, Sanman E, Schwebel DC, Segui-Gomez M, Shepard DS, Singh D, Singleton J, Sliwa K, Smith E, Steer A, Taylor JA, Thomas B, Tleyjeh IM, Towbin JA, Truelsen T, Undurraga EA, Venketasubramanian N, Vijayakumar L, Vos T, Wagner GR, Wang M, Wang W, Watt K, Weinstock MA, Weintraub R, Wilkinson JD, Woolf AD, Wulf S, Yeh PH, Yip P, Zabetian A, Zheng ZJ, Lopez AD, Murray CJ, AlMazroa MA, Memish ZA (2012). Global and regional mortality from 235 causes of death for 20 age groups in 1990 and 2010: a systematic analysis for the Global Burden of Disease Study 2010. Lancet.

[2] Rein DB, Stevens GA, Theaker J, Wittenborn JS, Wiersma ST (2012). The global burden of hepatitis E virus genotypes 1 and 2 in 2005. Hepatology.

[3] Gurley ES, Halder AK, Streatfield PK, Sazzad HM, Huda TM, Hossain MJ, Luby SP (2012). Estimating the burden of maternal and neonatal deaths associated with jaundice in Bangladesh: possible role of hepatitis E infection. Am J Public Health.

[4] Shah R, Nahar Q, Gurley ES (2016). One in five maternal deaths in Bangladesh associated with acute jaundice: results from a National Maternal Mortality Survey. Am J Trop Med Hyg.

[5] Teshale EH, Howard CM, Grytdal SP, Handzel TR, Barry V, Kamili S, Drobeniuc J, Okware S, Downing R, Tappero JW, Bakamutumaho B, Teo CG, Ward JW, Holmberg SD, Hu DJ (2010). Hepatitis E epidemic, Uganda. Emerg Infect Dis.

[6] Centers for Disease Control and Prevention (CDC) (2013). Investigation of hepatitis E outbreak among refugees: Upper Nile, South Sudan, 2012–2013. Morb Mortal Wkly Rep.

[7] Guthmann J-P, Klovstad H, Boccia D, Hamid N, Pinoges L, Nizou JY, Tatay M, Diaz F, Moren A, Grais RF, Ciglenecki I, Nicand E, Guerin PJ (2006). A large outbreak of hepatitis E among a displaced population in Darfur, Sudan, 2004: the role of water treatment methods. Clin Infect Dis.

[8] Zhu F-C, Zhang J, Zhang X-F, Zhou C, Wang ZZ, Huang SJ, Wang H, Yang CL, Jiang HM, Cai JP, Wang YJ, Ai X, Hu YM, Tang Q, Yao X, Yan Q, Xian YL, Wu T, Li YM, Miao J, Ng MH, Shih JW, Xia NS (2010). Efficacy and safety of a recombinant hepatitis E vaccine in healthy adults: a large-scale, randomised, double-blind placebo-controlled, phase 3 trial. Lancet.

[9] World Health Organization (2015). Hepatitis E vaccine: WHO position paper. Wkly Epidemiol Rec.

[10] Boccia D, Guthmann J-P, Klovstad H, Hamid N, Tatay M, Ciglenecki I, Nizou JY, Nicand E, Guerin PJ (2006). High mortality associated with an outbreak of hepatitis E among displaced persons in Darfur, Sudan. Clin Infect Dis.

[11] Browne LB, Menkir Z, Kahi V, Maina G, Asnakew S, Tubman M, Elyas HZ, Nigatu A, Dak D, Maung UA, Nakao JH, Bilukha O, Shahpar C, Centers for Disease Control and Prevention (CDC) (2015). Notes from the field: hepatitis E outbreak among refugees from South Sudan: Gambella, Ethiopia, April 2014–January 2015. Morb Mortal Wkly Rep.

[12] Phillips RM, Vujcic J, Boscoe A, Handzel T, Aninyasi M, Cookson ST, Blanton C, S Blum L, Ram PK (2015). Soap is not enough: handwashing practices and knowledge in refugee camps, Maban County, South Sudan. Confl Health.

[13] Siddiqui R (2015). South Sudan, Health Risks Increasing for People in Bentiu Protection of Civilian Camp.

[14] Iyer AS, Bouhenia M, Rumunu J, Abubakar A, Gruninger RJ, Pita J, Lino RL, Deng LL, Wamala JF, Ryan ET, Martin S, Legros D, Lessler J, Sack DA, Luquero FJ, Leung DT, Azman AS (2016). Immune responses to an oral cholera vaccine in internally displaced persons in South Sudan. Sci Rep.

[15] CCCM Cluster (2015). UN House PoC 3 Site Profile: June 2015.

[16] Lumley T (2004). Analysis of complex survey samples. J Stat Softw.

[17] Agresti A (2013). Categorical Data Analysis.

[18] Anderson RM, May RM (1992). Infectious Diseases of Humans: Dynamics and Control.

[19] Salje H, Cauchemez S, Alera MT, Rodriguez-Barraquer I, Thaisomboonsuk B, Srikiatkhachorn A, Lago CB, Villa D, Klungthong C, Tac-An IA, Fernandez S, Velasco JM, Roque VG, Nisalak A, Macareo LR, Levy JW, Cummings D, Yoon IK (2016). Reconstruction of 60 years of chikungunya epidemiology in the Philippines demonstrates episodic and focal transmission. J Infect Dis.

[20] Imai N, Dorigatti I, Cauchemez S, Ferguson NM (2015). Estimating dengue transmission intensity from sero prevalence surveys in multiple countries. PLoS Negl Trop Dis.

[21] McCarthy MC, He J, Hyams KC, el Tigani A, Khalid IO, Carl M (1994). Acute hepatitis E infection during the 1988 floods in Khartoum, Sudan. Trans R Soc Trop Med Hyg.

[22] Gelman A, Carlin JB, Stern HS, Dunson DB, Vehtari A, Rubin DB (2013). Bayesian Data Analysis.

[23] Kim J-H, Jong-Hoon K, Nelson KE, Panzner U, Kasture Y, Labrique AB, Wierzba TF (2014). A systematic review of the epidemiology of hepatitis E virus in Africa. BMC Infect Dis.

[24] Izopet J, Labrique AB, Basnyat B, Dalton HR, Kmush B, Heaney CD, Nelson KE, Ahmed ZB, Zaman K, Mansuy JM, Bendall R, Sauné K, Kamar N, Arjyal A, Karkey A, Dongol S, Prajapati KG, Adhikary D (2015). Hepatitis E virus seroprevalence in three hyperendemic areas: Nepal, Bangladesh and southwest France. J Clin Virol.

[25] Zhang J, Zhang X-F, Zhou C, Wang ZZ, Huang SJ, Yao X, Liang ZL, Wu T, Li JX, Yan Q, Yang CL, Jiang HM, Huang HJ, Xian YL, Shih JW, Ng MH, Li YM, Wang JZ, Zhu FC, Xia NS (2014). Protection against hepatitis E virus infection by naturally acquired and vaccine-induced immunity. Clin Microbiol Infect.

[26] Koshy A, Grover S, Hyams KC, Shabrawy MA, Pacsa A, al-Nakib B, Zaidi SA, al-Anezi AA, al-Mufti S, Burans J, Carl M, Richards AL (1996). Short-term IgM and IgG antibody responses to hepatitis E virus infection. Scand J Infect Dis.

[27] Favorov MO, Fields HA, Purdy MA, Yashina TL, Aleksandrov AG, Alter MJ, Yarasheva DM, Bradley DW, Margolis HS (1992). Serologic identification of hepatitis E virus infections in epidemic and endemic settings. J Med Virol.

[28] Favorov MO, Khudyakov YE, Mast EE, Yashina TL, Shapiro CN, Khudyakova NS, Jue DL, Onischenko GG, Margolis HS, Fields HA (1996). IgM and IgG antibodies to hepatitis E virus (HEV) detected by an enzyme immunoassay based on an HEV-specific artificial recombinant mosaic protein. J Med Virol.

[29] Pas SD, Streefkerk RHRA, Pronk M, de Man RA, Beersma MF, Osterhaus AD, van der Eijk AA (2013). Diagnostic performance of selected commercial HEV IgM and IgG ELISAs for immunocompromised and immunocompetent patients. J Clin Virol.

[30] Hoofnagle JH, Nelson KE, Purcell RH (2012). Hepatitis E. N Engl J Med.

[31] Zhang J, Zhang X-F, Huang S-J, Wu T, Hu YM, Wang ZZ, Wang H, Jiang HM, Wang YJ, Yan Q, Guo M, Liu XH, Li JX, Yang CL, Tang Q, Jiang RJ, Pan HR, Li YM, Shih JW, Ng MH, Zhu FC, Xia NS (2015). Long-term efficacy of a hepatitis E vaccine. N Engl J Med.

[32] Wickham H (2009). ggplot2: Elegant Graphics for Data Analysis.

