# Supplementary material for: High Hepatitis E Seroprevalence Among Displaced Persons in South Sudan
Source: Am J Trop Med Hyg. 2017 Jun 7;96(6):1296–301. doi: 10.4269/ajtmh.16-0620 (PMC5462562; doi:10.4269/ajtmh.16-0620)
Supplement: Supplementary file 1 [file SD1.pdf]

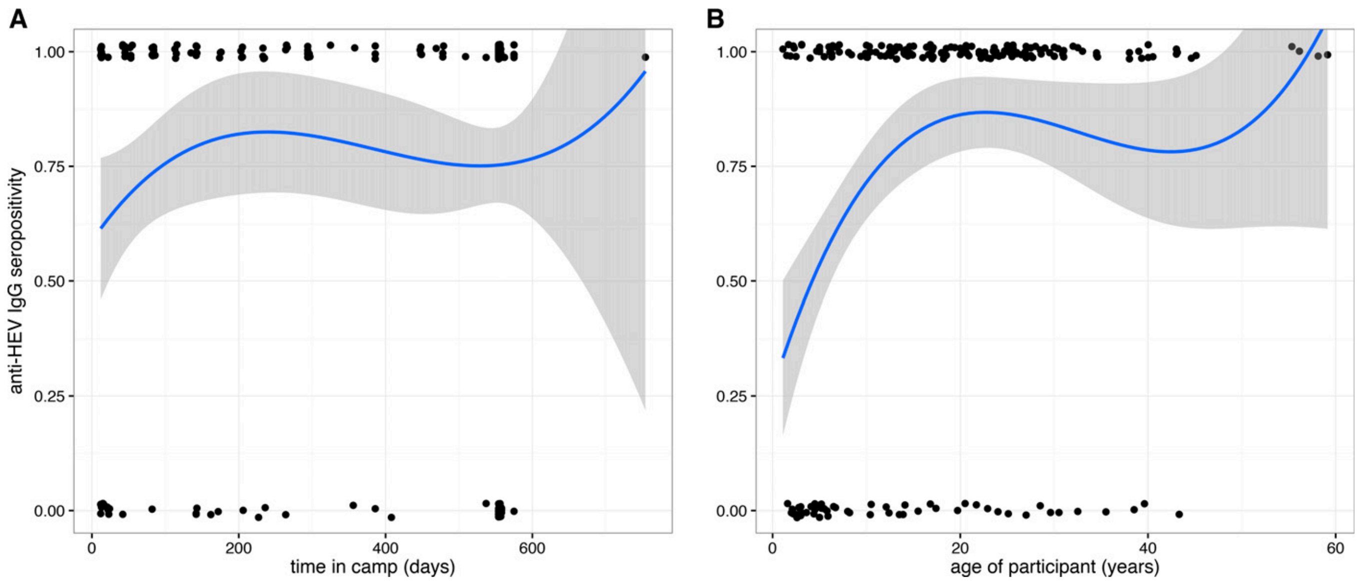

Supplemental Figure 1. Anti-hepatitis E virus (HEV) IgG seropositivity by (**A**) time in camp and (**B**) age. Blue line represents smoothed (basis splines with 3 degrees of freedom) estimate of proportion seropositive with the grey envelopes representing 95% confidence interval estimates from *geom_smooth()* in ggplot2 package of R.[32] Points represent positive (1) and negative (0) samples and are jittered for better visual display.

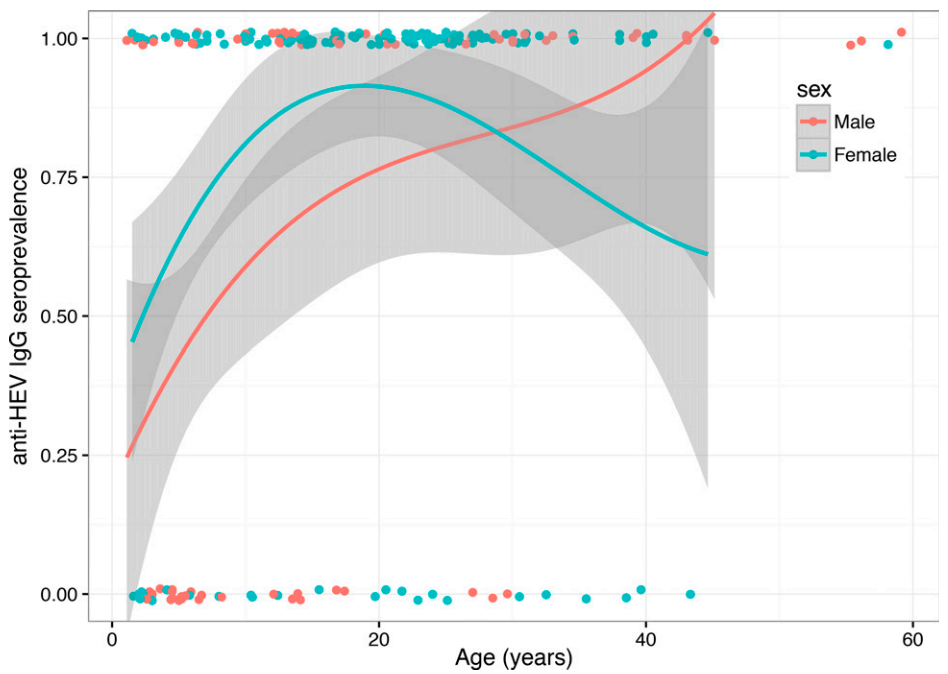

Supplemental Figure 2. Anti-hepatitis E virus (HEV) IgG seropositivity by age (*x*-axis) and sex (color). Lines represent smoothed (basis splines with 3 degrees of freedom) estimates of proportion seropositive with the grey envelopes representing 95% confidence interval estimates as estimated by *geom_smooth()* in ggplot2 package of R.[32] Points represent positive (1) and negative (0) samples and are jittered for better visual display and smoothed lines excluded high-leverage points at the upper end of the *x*-axis.

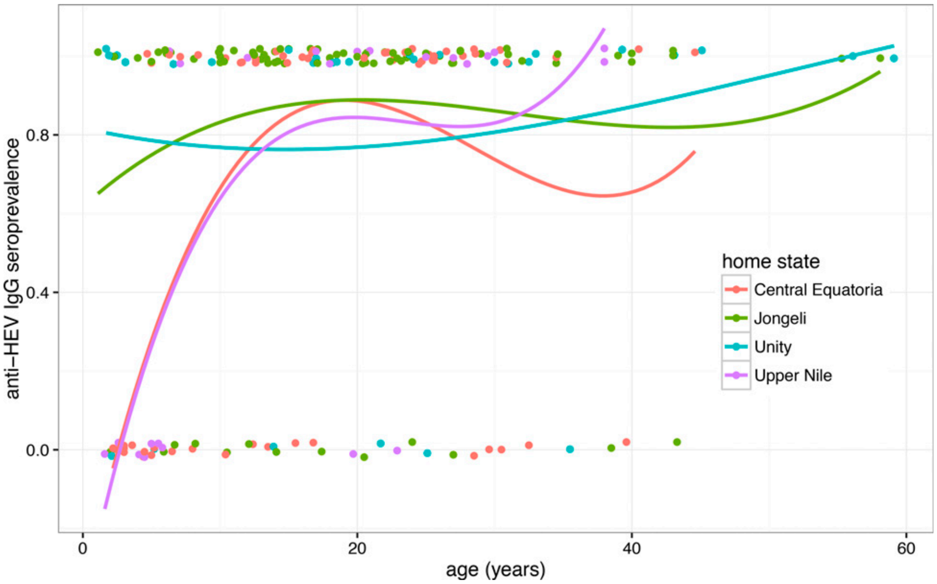

SUPPLEMENTAL FIGURE 3. Anti-hepatitis E virus (HEV) IgG seropositivity by age (*x*-axis) and home state (color). Lines represent smoothed (basis splines with 3 degrees of freedom) estimates of proportion seropositive with the grey envelopes representing 95% confidence interval estimates as estimated by *geom_smooth()* in ggplot2 package of R.[32] Points represent positive (1) and negative (0) samples and are jittered for better visual display.

SUPPLEMENTAL TABLE 1

Hazard ratios comparing the risk within the camp to the risk within individuals' home states assuming different times at risk within individuals home states (t_{max})

t_{max} (years)	Hazard ratio	95% Credible interval
15	1.2	0.1–3.3
20	1.8	0.2–4.5
25	2.3	0.3–5.8
30	2.8	0.3–6.7
35	3.0	0.5–7.1
40	3.2	0.6–7.4

SUPPLEMENTAL TABLE 2

Number and proportion positive to HEV IgG by age and sex category

	Anti-HEV IgG positive		
	n (proportion)		
Age class (years)	Males	Females	Males and females
1–4	5/15 (33%)	8/16 (50%)	13/31 (42%)
5–9	6/13 (46%)	10/12 (83%)	16/25 (64%)
10–19	15/21 (71%)	33/38 (87%)	48/59 (81%)
20–29	10/13 (77%)	40/45 (89%)	50/58 (86%)
30–39	6/6 (100%)	11/16 (69%)	17/22 (77%)
40+	7/7 (100%)	3/4 (75%)	10/11 (91%)
All ages	49/75 (65%)	105/131 (80%)	154/206 (75%)

HEV = hepatitis E virus.
